# Central nervous system effects of TAK-653, an investigational alpha-amino-3-hydroxy-5-methyl-4-isoxazole receptor (AMPAR) positive allosteric modulator in healthy volunteers

**DOI:** 10.1038/s41398-022-02148-w

**Published:** 2022-09-24

**Authors:** Francis Dijkstra, Patricio O’Donnell, Erica Klaassen, Derek Buhl, Mahnaz Asgharnejad, Laura Rosen, Rob Zuiker, Joop van Gerven, Gabriël Jacobs

**Affiliations:** 1 Centre for Human Drug Research (CHDR), Leiden, The Netherlands; 2 Department of Psychiatry, Leiden University Medical Center (LUMC), Leiden, The Netherlands; 3 Neuroscience Translational Medicine, Takeda Pharmaceutical Company Ltd, Cambridge, MA USA; 4 McLean Hospital, Department of Psychiatry, Harvard Medical School, Belmont, MA USA; 5 Neuroscience Therapeutic Area Unit, Takeda Pharmaceutical Company Ltd, Cambridge, MA USA

**Keywords:** Clinical pharmacology, Neuroscience

## Abstract

TAK-653 is a novel AMPA receptor positive allosteric modulator in clinical development for the treatment of major depressive disorder (MDD). This study aimed to measure the functional pharmacodynamic central nervous system (CNS) effects of TAK-653. A randomised, double-blind, placebo-controlled, three-way crossover (placebo, TAK-653 0.5 mg and 6 mg) study with 24 healthy volunteers was performed. NeuroCart tests consisting of body sway (BS), saccadic peak velocity (SPV), smooth pursuit eye movements (SP), adaptive tracking (AT), Bowdle and Bond and Lader Visual Analogue Scales (B-VAS and BL-VAS) and Stroop test were performed pre-dose and 3.5 and 4 h post-dose. Data were analysed using a mixed model analysis of covariance with baseline as covariate. It was found that TAK-653 did not affect BS and subjective drug effects as measured by B-VAS and BL-VAS at either dose level. TAK-653 0.5 mg increased SPV (degrees/second) (19.49 [5.98, 32.99], *P* = 0.02) and affected Stroop difference in reaction time between correct congruent and correct incongruent answers and number of correct responses in incongruent trials (22.0 [4.0, 40.0], *P* = 0.05 and −0.3 [−0.5, −0.1], *P* = 0.02, respectively). TAK-653 6 mg improved AT (%) (1.68 [0.51, 2.84], *P* = 0.02) and increased SPV (degrees/s) (15.40 [1.91, 28.90], *P* = 0.06) and SP (%) (2.32 [0.37, 4.27], *P* = 0.05). Based on these findings it can be concluded that TAK-653 demonstrated a psychostimulant-like pharmacodynamic profile on the NeuroCart consistent with previously reported increase of cortical excitability following Transcranial Magnetic Stimulation (TMS) of the human motor cortex.

## Introduction

Since ketamine, a N-methyl-D-aspartate (NMDA) receptor antagonist, has been shown to have rapid occurring antidepressant effects [1–3], there is growing interest in the NMDA receptor as potential novel target for the pharmacological treatment of depressive disorders. Studies into the mechanisms underlying the antidepressant effects of NMDA receptor antagonism have demonstrated an important role for alpha-amino-3-hydroxy-5-methyl-4-isoxazole (AMPA) receptor-mediated signalling [4–7]. Blocking NMDA receptors and thereby indirectly stimulating AMPA receptors, leads to a shift towards predominantly stimulatory glutamate-mediated neurotransmission [4–7]. This is believed to affect molecular processes implicated in the pathophysiology of (chronic) mood disorders related to synaptic plasticity and/or cellular resilience [8], including the enhanced production of brain-derived neurotrophic factor and triggering of the mammalian target of rapamycin (mTOR) signalling [6]. The importance of AMPA receptor-mediated signalling is further supported by the finding that the preclinical antidepressant-like effects of ketamine and related compounds are opposed by AMPA receptor antagonists [9]. These findings support the development of novel antidepressants that target AMPA receptors.

The novel AMPA receptor positive allosteric modulator (PAM) TAK-653 (9-[4-(cyclohexyloxy)phenyl]-7-methyl-3,4-dihydropyrazino[2,1-*c*][1,2,4] thiadiazine 2,2-dioxide) is an investigational potential therapeutic compound in clinical development for major depressive disorder. As full functional agonism of AMPA receptors is associated with potential untoward central nervous system (CNS) stimulation, AMPA receptor PAMs have been proposed as an alternative pharmacological strategy for glutamatergic modulation [10]. In initial healthy volunteer studies with oral doses of TAK-653 0.3 mg to 18 mg, the compound was well tolerated and, in contrast to ketamine, did not cause dissociative adverse effects [11]. Maximum plasma concentrations were attained within 1.25 h to 5 h after dosing, the terminal half-life varied from 33.1 h to 47.8 h and cerebrospinal fluid concentrations were suggestive of rapid brain penetration [11]. These pharmacokinetic (PK) and safety profiles in healthy volunteers were promising for further clinical development, but the pharmacodynamic (PD) properties of TAK-653 had not been systematically assessed.

In early phases of clinical drug development a full characterisation of both the PK and PD properties of innovative compounds is crucial to rationally guide drug development [12, 13]. The question-based clinical development (QBCD) concept has previously been proposed as a conceptual framework for characterising drugs in early clinical development [12]. Specific to CNS drug development, QBCD allows for systematic investigation of crucial issues such as blood–brain barrier (BBB) penetration, intended target engagement and off-target effects [12]. By explicitly incorporating methodologies to address these issues when designing early-phase CNS studies, findings may support go/no-go decisions in subsequent development phases [12].

In order to characterize TAK 653's PD profile, we applied transcranial magnetic stimulation (TMS) as a potential biomarker for cortical excitability and we performed a test battery of extensively validated, drug-sensitive neurophysiological and neurocognitive CNS tests, the Neurocart [14]. Based on its in vitro profile and preclinical effects, TAK-653 was hypothesised to yield stimulatory CNS effects in healthy clinical populations. As TAK-653 was the first AMPA receptor PAM to be tested using the NeuroCart it was decided to compare the NeuroCart profile of TAK-653 to the profiles of both excitatory or CNS-stimulant (e.g. dopamine releasers) [15] and inhibitory or CNS-depressant (e.g. GABA_A_-agonists) compounds [16–21]. Although the mechanism of action of these compounds differs from TAK-653's mechanism of action, their PD profiles were expected to be relevant for the ‘pharmacological benchmarking’ of TAK-653's functional PD effects in healthy humans.

Our previous paper reported the effects of TAK-653 0.5 mg and 6 mg on TMS motor evoked potentials following stimulation of the motor cortex [22]. It was observed that TAK-653 increased the amplitude of motor evoked potentials, indicative of increased AMPA receptor-mediated cortical excitability [22]. In the current paper, the NeuroCart data will be presented.

## Methods

### Study design and participants

**Fig. 1:**
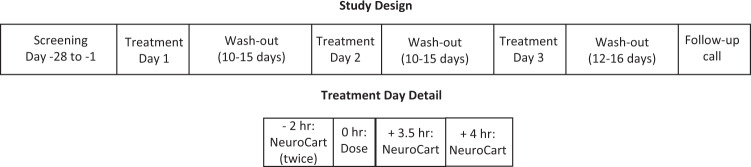
Study design. Study design and the timing of NeuroCart assessments on treatment days.

The study was a randomised, double-blind, placebo-controlled, three-period crossover study (Fig. 1). A fourth, open-label period was conducted with ketamine for assay sensitivity, but the TMS and NeuroCart results were equivocal for reasons discussed previously [22], and will not be discussed here. During the three-period crossover phase, each treatment period was one day in duration and separated by a wash-out period of 10 to 15 days. During treatment days, volunteers received one oral dose of either placebo, TAK-653 0.5 mg or TAK-653 6 mg; all treatments had the same appearance to guarantee blinding of volunteers and research staff. At 12 to 16 days after the last study visit, a telephone call was made to volunteers as part of the follow-up procedure.

TAK-653 dose levels of 0.5 mg and 6 mg were selected as the lower dose was expected to have minimum to no effects based on the preclinical data and the higher dose to fall into the pharmacological active range [22]. Both dose levels were well tolerated in the previous healthy volunteer study [11].

Twenty-four healthy volunteers were included in this study. To assess eligibility, volunteers were screened using the following procedures: a review of their medical and psychiatric history, a physical examination, measurement of vital signs, an electrocardiogram (ECG), blood chemistry and haematology laboratory assessments, and urinalysis. Volunteers who had a clinically significant previous or current psychiatric disorder according to Diagnostic and Statistical Manual-5 were excluded. In addition, volunteers who had a history of drinking an average of two or more alcoholic drinks per day were excluded.

During treatment days, volunteers reported at the research facility in the morning. Before a test dose was given, safety assessments were performed, including: adverse event (AE) occurrence, a physical examination, a suicidality assessment using the Columbia suicide severity rating scale [23], measurement of vital signs, an ECG, laboratory assessments and urinalysis. The same safety assessments and AE recording were performed at set times after dosing.

During the study, volunteers were instructed to restrict the use of substances that could alter brain activity, including concomitant medication, alcoholic beverages, caffeinated products and nicotine-containing products. First, volunteers were instructed not to use concomitant medication starting from the seven days before the first test dose through to the end of the study. Second, they were instructed not to consume alcoholic beverages seven days before the screening visit and each treatment day. Third, they were instructed not to consume caffeinated products 24 h before the screening visit and each treatment day. Outside of these restrictions, volunteers could consume up to six servings of caffeinated products a day. Finally, they were instructed not to use nicotine-containing products 48 h before the screening visit and each treatment day. Otherwise, volunteers could use up to five nicotine-containing products per day.

### Pharmacokinetic assessments

The PK sample collection times were aligned with timings of the TMS assessments, as these were the assessments of primary interest in this study; PK samples were collected before drug administration, and 0.5 h and 2.5 h after administration. Based on initial healthy volunteer studies, it was expected that the mean maximal plasma concentration (C_max_) would be reached at 2.5 h post-dose and due to the relatively long terminal half-life (t_1/2_) TAK-653 of 33.1 to 47.8 h, plasma levels were then expected to remain stable over a few hours [11]. A validated high-performance liquid chromatography with tandem mass spectrometry assay with a lower limit of quantification of 0.1 ng/mL and coefficient of variation between 1.41% and 5.22% was used to measure TAK-653's plasma concentrations.

### Functional pharmacodynamic NeuroCart assessments

NeuroCart tests that have been shown sensitive to CNS depressant and/or CNS stimulant compounds were selected for this study [14]. These were: body sway, smooth pursuit eye movements, saccadic eye movements, adaptive tracking test, Stroop coloured word test, and Bond and Lader and Bowdle Visual Analogue Scales (VAS). In Table 1 the effects of different CNS depressant and CNS stimulant compounds on the NeuroCart tests performed in this study are summarised. The tests were performed twice prior to dosing as well as at the time of expected maximum plasma concentrations, namely 3.5 hours and 4 hours post-dose (Fig. 1). During all tests, lighting conditions were standardised and volunteers were comfortably seated in front of a computer screen, except for body sway measurements, for which volunteers were standing.

**Table 1:** Summary of effects of CNS depressant and CNS stimulant compounds on selected NeuroCart tests.

Test	CNS depressant		CNS stimulant			
	Diazepam 10 mg	Benzodiazepines (dose unspecified)	Modafinil 200 mg	Dexamphetamine 20 mg	Methylphenidate (average 20 mg)	Caffeine (60 mg)
Body sway (%)	+^a^	NR^c^	–	–	–	NR
Smooth pursuit (%)	-^b^	NR	NR	NR	+	NR
Saccadic peak velocity (deg/s)	NR	–	+	+	NR	+
Adaptive tracking (%)	NR	–	+	+	+	+
Stroop coloured word test	NR	–	NR	NR	NR	NR
Bond and Lader VAS alertness	NR	–	+	+	+	+
Bowdle VAS Feeling high	No effect	No effect	No effect	+	No effect	No effect

^a^+indicates improvement or increase; ^b^- indicates deterioration or decrease; ^c^*NR* not reported

#### Body sway

Body sway measurements are used to assess postural stability and are often used in pharmacologic studies [24–26]. Measurements of movements in the anteroposterior direction were performed as in previously published studies [26, 27], with a string similar to the Wright ataxiameter [28] attached to the waist of participants. Volunteers were instructed to stand comfortably on a firm surface with their feet slightly apart and eyes closed for 2 minutes. In previous studies, CNS-stimulant compounds demonstrated reductions in body sway; for example, modafinil (200 mg), dexamphetamine (20 mg) and clinical doses (average 20 mg) of methylphenidate reduced body sway by approximately 35% [29], 19.4% [15] and 36.8%, respectively [30]. Conversely, CNS-depressant compounds such as benzodiazepines are associated with increased body sway; for example, diazepam (10 mg) increased body sway by 119% [31].

#### Smooth pursuit eye movements

The computerised smooth pursuit measurement was performed as described previously [32] and used in many studies to assess drug effects [14]. During this test, participants followed a light source with their eyes, that moved continuously in a horizontal direction on a screen placed 58 cm away. The outcome of smooth pursuit was defined as the percentage of time the participant’s eyes were in smooth pursuit of the target for each stimulus velocity and frequency. In a previous study, the velocity of smooth pursuit eye movements was impaired by diazepam (10 mg) [32]. Improvements in smooth pursuit of approximately 6% have been reported with CNS stimulants such as methylphenidate (average 20 mg) [30].

#### Saccadic eye movements

The computerised measurement of saccadic eye movements was performed as described previously [33] and used in many pharmacological studies [14]. Briefly, to measure saccadic eye movements, participants were positioned identically to when performing the smooth pursuit measurement and instructed to follow a light source that jumped from side to side [33]. The parameter collected was saccadic peak velocity (SPV) in degrees/second (deg/s). Previous studies have demonstrated that CNS-stimulant compounds such as caffeine (60 mg), modafinil (200 mg) and dexamphetamine (20 mg) increased the average SPV by 11.6 deg/s [34], 24.6 deg/s [29], and 12.7 deg/s [15], respectively. CNS-depressant compounds such as benzodiazepines have been shown to decrease SPV [35].

#### Adaptive tracking test

Adaptive tracking tests have been used in many pharmacological studies to evaluate visuomotor coordination and vigilance [14]. In this study, we used an adaptive tracking test according to specifications from Borland and Nicholson [36]. During the test, a circle moved randomly on a screen. Participants were given a joystick and instructed to use it to keep a dot within the moving circle. When an effort was successful, the speed of the moving circle increased. Conversely, the speed decreased if the participant was not able to maintain the dot within the circle, resulting in a constant and individually adapted challenge throughout the procedure. The outcome of the test is the average speed of the moving circle as a percentage of the maximum speed of the circle. In previous studies, CNS-stimulant compounds such as caffeine (60 mg), modafinil (200 mg), dexamphetamine (20 mg) and methylphenidate (average 20 mg) improved average adaptive tracking by approximately 1.6% [34], 1.8% [29], 4.2% [15], and 2.2% [30], respectively. For CNS-depressant compounds, such as benzodiazepines, an impairment of adaptive tracking has been demonstrated [14].

#### Stroop coloured word test

The Stroop effect test involves identifying the colour of coloured words [37], many CNS-active compounds have an effect on this test [38–40]. In this study, we used a computer-adapted version from the Psychology Software Tools website (https://pstnet.com/products/e-prime/), comprising two subtests as described in a previous publication [41]. In the first subtest, six coloured items were presented at random. The possible colours were green, red and blue, and each colour corresponded to a number key on the numpad section of the keyboard; green corresponded with 1, red with 2 and blue with 3. Participants were instructed to place the index, middle and ring fingers of their dominant hand on keys 1, 2 and 3. When a coloured item appeared on the screen, participants were to press the corresponding key as quickly as possible. In the second subtest, which immediately followed the first, 34 colour and word pairs were presented randomly. The words that were used were ‘red’, ‘green’ and ‘blue’, and the colour and word pairs were either congruent or incongruent matches. Again, the participants were asked to identify the correct colour as quickly as possible by pressing either keys 1, 2 or 3 on the numpad. Each item or word was shown for 4 seconds, and there was a 0.5 s pause after every response. Two parameters were derived from this test: Stroop 1 is the difference in reaction time between correct congruent and correct incongruent answers (ms) and Stroop 2 is the number of correct responses in incongruent trials. Previous studies demonstrated that benzodiazepines impair performance on this test [38].

#### Bond and Lader and Bowdle Visual Analogue Scales

VAS, as originally described by Norris, have commonly been used to quantify the subjective effects of sedative agents [35, 42]. Subjects were instructed to use the computer mouse to select their response to each VAS item. The Bond and Lader VAS involved collecting scores from 16 horizontal scales related to how a person feels. From these measurements, three main factors, namely ‘alertness’, ‘mood’ and ‘calmness’, were calculated as described in previous publications [34, 43]. Benzodiazepines have consistently shown reductions on VAS alertness [44], whereas variable but consistent increases are observed with caffeine [34], dexamphetamine [15], modafinil [29], and methylphenidate [30]. Psychedelic effects were measured using the Bowdle VAS as previously described [45]. This scale consists of 13 items on which three summary scales (internal perception, external perception and ‘feeling high’) are calculated using log transformation as described in previous publications [46]. Dexamphetamine (20 mg) has been shown to increase the summary scale ‘feeling high’ [15], in contrast to the other CNS stimulants mentioned earlier.

### Statistical analysis

Analyses were performed using SAS software version 9.4 (SAS Institute, Cary, NC, USA). Residual Q-Q plots were produced for all NeuroCart parameters to check the assumption of normality of the error term in the mixed effects models. This was done by visual inspection and the Shapiro–Wilk test statistic. To assess the treatment effects, data for each parameter were analysed with a mixed model analysis of covariance. We defined treatment, time, period and treatment by time as fixed factors; subject, subject by treatment and subject by time as random factors; and the (average) baseline measurement per study period as a covariate. The Kenward–Roger approximation was used to estimate denominator degrees of freedom and the model parameters were estimated using the restricted maximum likelihood method. Individual treatment effects over the 4-hour post-dose time period for the different doses were reported with the least squares mean estimated difference, the two-sided 90% confidence interval (CI) and the *P*-value. Owing to the exploratory nature of this study, a 90% CI instead of 95% CI was deemed sufficient. Next to that, no correction for multiple comparisons was performed as due to the exploratory nature of this study, hypothesis testing was not used in the strict way, but to guide the direction of future research.

## Results

### Demographics

**Fig. 2:**
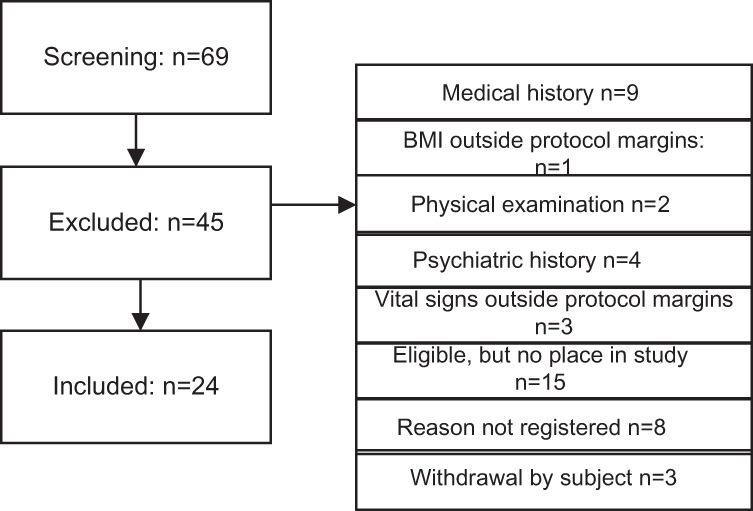
Subject disposition.

In total, 69 volunteers were screened, of which 24 healthy volunteers (23 male and 1 female of non-childbearing potential) between 18 and 55 years of age were included (Table 2). Subject disposition can be found in Fig. 2. All participants completed the three study periods.

**Table 2:** Demographic characteristics of study participants.

Characteristic	Subjects enrolled (*N* = 24)
Age (years), mean (SD^a^)	27.9 (9.0)
Sex, *n* (%)	
Female	1 (4.2%)
Male	23 (95.8%)
Weight (kg), mean (SD)	79.12 (10.81)
Height (cm), mean (SD)	181.98 (9.88)
BMI^b^(kg/m^2^), mean (SD)	23.92 (2.85)

^*a*^*SD* standard deviation; ^b^*BMI* body mass index

### Pharmacokinetic assessments

As reported in our previous publication, mean (SD) TAK-653 plasma levels for the 0.5 mg dose were 0.99 (0.94) ng/ml at 0.5 h post-dose and 4.19 (0.83) ng/ml at 2.5 h post-dose [22]. Plasma levels for the 6 mg dose were 2.57 (3.29) ng/ml at 0.5 h post-dose and 45.99 (8.84) ng/ml at 2.5 h post-dose [22].

### Functional pharmacodynamic NeuroCart assessments

**Fig. 3:**
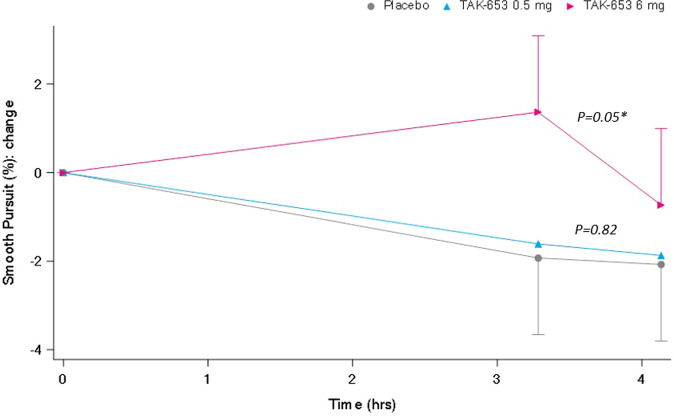
Smooth pursuit eye movements. Smooth pursuit eye movements: change from baseline time effect profile of the least square (LS) mean (90%CI).

**Fig. 4:**
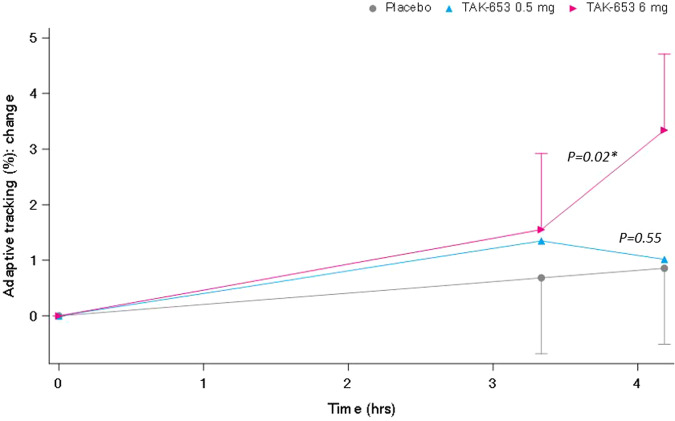
Adaptive tracking. Adaptive tracking: change from baseline time effect profile of the least square (LS) mean (90%CI).

**Fig. 5:**
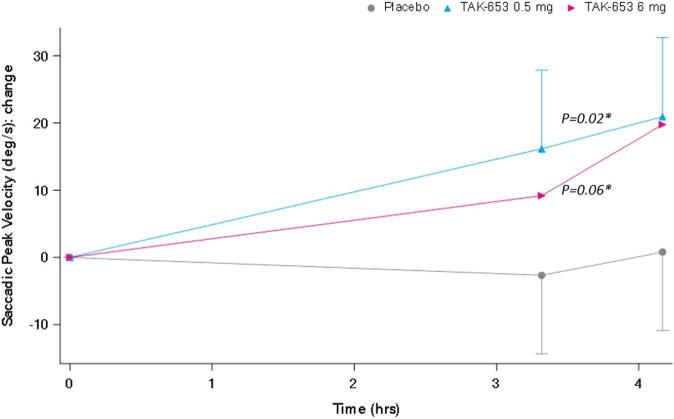
Saccadic peak velocity. Saccadic peak velocity: change from baseline time effect profile of the least square (LS) mean (90%CI).

All NeuroCart parameters were normally distributed, except body sway measurements, which were log-normal distributed and therefore natural log transformation was applied for their analysis. For interpretation back transformation was applied. To calculate summary scores for VAS Bowdle, log transformation was performed as well as described in previous publications [46]. Results are summarised in Table 3. On smooth pursuit eye movements (%), a clear statistically significant improvement was observed with the TAK-653 6 mg dose (Fig. 3). At the same dose level, a similar improvement was observed for adaptive tracking (%) (Fig. 4). Both doses of TAK-653 increased SPV (deg/s) to a similar extent (Fig. 5).

**Table 3:** Least squares mean overall treatment effects and individual treatment effects of TAK-653 0.5 mg and 6 mg over the 4-hour post-dose period.

	Least squares mean			Contrasts (90% CI) *P*-value	
	Placebo	TAK-653 0.5 mg	TAK-653 6 mg	TAK-653 0.5 mg vs. placebo Estimate of difference, 90% CI^a^, *p*-value	TAK-653 6 mg vs. placebo Estimate of difference, 90% CI^a^, *p*-value
Body sway, log (mm) (*N* = 24)	202.3	199.6	185.7	−1.3% (−13.4%, 12.4%) *P* = 0.86	−8.2% (−19.4%, 4.6%) *P* = 0.28
Smooth pursuit (%) (*N* = 24)	44.5	44.8	46.8	0.26 (−1.69, 2.21) *P* = 0.82	2.32 (0.37, 4.27) *P* = 0.05*
Saccadic peak velocity (deg/s) (*N* = 24)	475.5	495.0	490.9	19.49 (5.98, 32.99) *P* = 0.02^*^	15.40 (1.91, 28.90) *P* = 0.06^*^
Adaptive tracking (%) (*N* = 24)	30.8	31.2	32.5	0.41 (−0.73, 1.56) *P* = 0.55	1.68 (0.51, 2.84) *P* = 0.02^*^
Stroop 1^c^ (ms) (*N* = 24)	71.4	93.4	71.0	22.0 (4.0, 40.0) *P* = 0.05^*^	−0.5 (−18.3, 17.3) *P* = 0.96
Stroop 2^d^ (ms) (*N* = 24)	16.7	16.4	16.6	−0.3 (−0.5, −0.1) *P* = 0.02^*^	−0.1 (−0.3, 0.2) *P* = 0.61
VAS^b^ alertness (mm) (*N* = 24)	49.9	50.5	50.7	0.65 (−0.38, 1.67) *P* = 0.30	0.77 (−0.24, 1.79) *P* = 0.21
VAS^b^ calmness (mm) (*N* = 24)	52.4	52.4	52.2	−0.03 (−1.65, 1.60) *P* = 0.98	−0.25 (−1.88, 1.38) *P* = 0.80
VAS^b^ mood (mm) (*N* = 24)	51.5	51.8	52.0	0.28 (−0.29, 0.85) *P* = 0.41	0.48 (−0.10, 1.05) *P* = 0.17
VAS^b^ external, log (mm) (*N* = 24)	0.35	0.35	0.35	−0.01 (−0.03, 0.01) *P* = 0.63	−0.01 (−0.03, 0.01) *P* = 0.58
VAS^b^ internal, log (mm) (*N* = 24)	0.35	0.35	0.35	< 0.00 (−0.01, 0.01) *P* = 0.92	<0.00 (−0.01, 0.02) *P* = 0.76
VAS^b^ ‘feeling high’, log (mm) (*N* = 24)	0.37	0.35	0.34	−0.02 (−0.05, 0.01) *P* = 0.25	−0.03 (−0.06, −0.01) *P* = 0.05^*^

^a^*CI* confidence interval, ^b^*VAS* Visual Analogue Scale; ^c^Stroop 1 is the difference in reaction time between correct congruent and correct incongruent answers; ^d^Stroop 2 is the number of correct responses in incongruent trials. ^*^Indicates a statistically significant effect.

The VAS Bowdle subscale ‘feeling high’ remained stable under TAK-653 0.5 mg and 6 mg; however, average VAS-high increased with placebo, resulting in a statistically significant reduction with TAK-653 6 mg. A review of the raw data revealed that this effect was caused by one subject who indicated a 20 mm (large) increase in VAS-high after placebo. This entry was judged to be an artefact, given ‘feeling high’ does not occur spontaneously or under placebo and the subject did not have AEs indicating subjective drug effects such as ‘feeling abnormal’, ‘feeling drunk’ or ‘feeling high’.

On the Stroop coloured word test TAK-653 0.5 mg increased the difference in reaction time between correct congruent and correct incongruent answers and decreased the number of correct responses in incongruent trials compared to placebo whereas TAK-653 6 mg did not affect any parameter of the Stroop coloured word test.

No significant effects were observed on body sway (%), other VAS Bowdle subscales (mm) or any of the Bond and Lader VAS subscales (mm) (Table 2).

### Safety and tolerability

For details on TAK-653's safety and tolerability in this study, please refer to previous reported results [22]. In summary, TAK-653 was well tolerated, no serious AEs were observed and there were no withdrawals related to an AE. The most frequently reported AEs after administration of TAK-653 were somnolence (TAK-653 0.5 mg: 3 of 24 subjects [12.5%], TAK-653 6 mg: 3 of 24 subjects [12.5%]), headache (TAK-653 0.5 mg: 1 of 24 subjects [4.2%], TAK-653 6 mg: 4 of 24 subjects [16.7%]) and nasopharyngitis (TAK-653 0.5 mg: 3 of 24 subjects [12.5%], TAK-653 6 mg 1 of 24 subjects [4.2%]). Of these AEs, somnolence and headache were reported after administration of placebo as well (2 of 24 subjects [8.3%] each). No clinically significant effects on vital signs, ECGs or laboratory measurements were observed. Of note is that no AEs of seizure, dissociative effects or euphoria were observed.

## Discussion

Similar to CNS stimulant compounds TAK-653 increased SPV, SP and adaptive tracking at the time maximum plasma concentration was reached. These effects were more pronounced with 6 mg than with 0.5 mg TAK-653. TAK-653 increased SPV at both 0.5 mg and 6 mg, while smooth pursuit eye movements and adaptive tracking increased at 6 mg but not at 0.5 mg. The effects of TAK-653 on the NeuroCart tests contrasted with the effects of CNS-depressant compounds such as benzodiazepines, which have been shown to decrease smooth pursuit eye movements [32], SPV [44], and adaptive tracking [14]. The absence of an effect of TAK-653 on any of the VAS subscales supports the finding that TAK-653 is devoid of subjective mood-related derangements observed with other CNS-stimulant compounds such as dexamphetamine [15]. When comparing the acute pharmacodynamic NeuroCart profile of TAK-653 to known profiles of CNS stimulant and CNS depressant compounds, TAK-653's profile is suggestive of stimulatory CNS effects. This is consistent with the TMS-EMG (electromyography) findings demonstrating increased cortical excitability with the 6 mg dose [22]. While one could argue that the observed effects on the NeuroCart tests are due to TMS itself, this can be ruled out for this study as a placebo arm was included and the effects of the different doses of TAK-653 on the NeuroCart were observed compared to placebo.

Compared to clinical doses of psychostimulants previously characterised using Neurocart test, TAK-653's stimulatory CNS effects appear more limited. TAK-653 increased SPV by 15.5 to 19.5 deg/s, which is larger than caffeine 60 mg (11.6 deg/s) [34] and dexamphetamine 20 mg (12.7 deg/s) [15], but smaller than modafinil 200 mg (24.6 deg/s) [29]. Increases in smooth pursuit eye movements represented only roughly one-third of those induced by methylphenidate [30]. The increase in adaptive tracking of 1.6% with TAK-653 6 mg was comparable to modafinil (1.8%) [29], caffeine (1.6%) [34], and methylphenidate (2.2%) [30], but smaller than dexamphetamine (4.2%) [15]. Next to that, decreases in BS have been observed for other CNS stimulant compounds, but no effect of both dose levels TAK-653 on BS was observed. Although direct comparisons should be made for an unequivocal interpretation of our findings, TAK-653 seems to have a novel stimulatory CNS profile that is generally more subtle than clinical doses of known psychostimulants, and distinguishes itself by a relatively large stimulatory effect on saccadic peak velocity but devoid of any subjective mood-related derangement such as dysphoria, anxiety or feeling high.

Although TAK-653 demonstrated psychostimulant effects, its impact on different aspects of cognition was less consistent. The Stroop test was included as it can be helpful in understanding complex attention, perception and elements of executive function [47]. TAK-653 0.5 mg but not 6 mg increased the difference in reaction time between correct congruent and correct incongruent answers. Similarly, TAK-653 0.5 mg but not 6 mg decreased the number of correct responses in incongruent trials. It cannot be excluded that the lower dose may affect aspects of cognitive functioning, which are obscured at a higher dose. The overall pharmacodynamic profile, however, provides no reason to assume a bell-shaped dose-response curve. Therefore, the Stroop results are currently best considered as a potential type I error of a less robust test with multiple complex endpoints.

The PK results of this study were in line with results from initial healthy volunteer studies, as mean maximal plasma concentrations for 0.5 and 6 mg TAK-653 were comparable to those observed at similar dose levels [11]. Therefore, although the area under the curve from time 0 to infinity (AUC_∞_) was not determined in the current study, this was expected to correspond to the AUC_∞_ observed in initial healthy volunteer studies with 406 h*ng/ml and 3167 h*ng/ml for TAK-653 0.5 and 6 mg, respectively. These data support dose and concentration dependence since more pronounced effects were observed with the 6 mg dose compared to the 0.5 mg dose.

A limitation of this study is that full dose/concentration-response characterisation was precluded by safety concerns. Given both AMPA receptor PAMs and TMS are associated with an increased albeit very limited risk of convulsions [10], 6 mg was selected as the highest dose as it was expected to yield a mean maximum plasma concentration well below those at which partial seizures were observed in primates (Takeda internal data).

Taken together, the PD profile of TAK-653 was characterised in this study according to recommendations by the “QBDD” framework [12]. As hypothesised based on its mechanism of action of AMPA receptor PAM, TAK-653 demonstrated an acute functional PD profile of CNS stimulatory effects on the NeuroCart. This confirms BBB penetration and, moreover, target engagement that is consistent with the previously reported TMS results of increased cortical excitability [22]. No undesired pharmacological effects associated with AMPA receptor stimulation, such as seizures or euphoria, off-target effects or unexpected AEs, were observed in this acute dosing study. The insights obtained in this study, can be used to design future studies in both healthy individuals and selected patient populations that are hypothesised to benefit from AMPA receptor-mediated stimulatory CNS effects.
